# Effects of Apoptin-Induced Endoplasmic Reticulum Stress on Lipid Metabolism, Migration, and Invasion of HepG-2 Cells

**DOI:** 10.3389/fonc.2021.614082

**Published:** 2021-02-26

**Authors:** Yilong Zhu, Yiquan Li, Bing Bai, Chao Shang, Jinbo Fang, Jianan Cong, Wenjie Li, Shanzhi Li, Gaojie Song, Zirui Liu, Jin Zhao, Xiao Li, Guangze Zhu, Ningyi Jin

**Affiliations:** ^1^ Academicians Workstation of Jilin Province, Changchun University of Chinese Medicine, Changchun, China; ^2^ Institute of Military Veterinary Medicine, Academy of Military Medical Science, Changchun, China; ^3^ Jiangsu Co-innovation Center for Prevention and Control of Important Animal Infectious Diseases and Zoonoses, Yangzhou University, Yangzhou, China

**Keywords:** Apoptin, endoplasmic reticulum stress, lipid metabolism, migration, invasion

## Abstract

In this study, we investigated the effects of Apoptin-induced endoplasmic reticulum (ER) stress on lipid metabolism, migration and invasion of HepG-2 cells, and preliminarily explored the relationship between endoplasmic reticulum stress, lipid metabolism, migration, and invasion. The effects of Apoptin on ER function and structure in HepG-2 cells were determined by flow cytometry, fluorescence staining and western blotting by assessing the expression levels of ER stress related proteins. The effects of Apoptin on HepG-2 cells’ lipid metabolism were determined by western blot analysis of the expression levels of triglyceride, cholesterol, and lipid metabolism related enzymes. The effects of Apoptin on HepG-2 cells’ migration and invasion were studied using migration and invasion assays and by Western-blot analysis of the expression of proteins involved in migration and invasion. The *in vivo* effects of endoplasmic reticulum stress on lipid metabolism, migration and invasion of HepG-2 cells were also investigated by immunohistochemistry analysis of tumor tissues from HepG2 cells xenografted nude mice models. Both *in vitro* and *in vivo* experiments showed that Apoptin can cause a strong and lasting ER stress response, damage ER functional structure, significantly change the expression levels of lipid metabolism related enzymes and reduce the migration and invasion abilities of HepG-2 cells. Apoptin can also affect HepG-2 cells’ lipid metabolism through endoplasmic reticulum stress and the abnormal expression of enzymes closely related to tumor migration and invasion. These results also showed that lipid metabolism may be one of the main inducements that reduce HepG-2 cells’ migration and invasion abilities.

## Introduction

Apoptin is derived from chicken anemia virus (1), which has two nuclear localization signals. These signals can help Apoptin to specifically enter tumor cells’ nuclei and interact with the APC1 subunit of APC/C (2, 3), causing a C2/M cell cycle arrest and tumor cell apoptosis (4, 5). Apoptin can also interact with the nuclear orphan receptor Nur77 to transfer the apoptotic signal from nucleus to mitochondria (6, 7), which induces the release of cytochrome c that activates Caspase-3, resulting in tumor cell apoptosis (8). At present, no relevant literature has reported the effect of Apoptin on the functional structure of the endoplasmic reticulum. In our previous studies, we found that Apoptin can affect this functional structure by causing a strong and lasting endoplasmic reticulum stress response.

The endoplasmic reticulum (ER) is the main site of protein, lipid and carbohydrate synthesis. It is essential for the maintenance of intracellular homeostasis and imbalance of the endoplasmic reticulum homeostasis can seriously affect its function (9). ER stress related proteins mainly include Protein Kinase like Endoplasmic Reticulum Kinase (PERK), Calnexin, Endoplasmic Reticulum Oxidoreduclin 1-Lα (Ero1-Lα), Protein Disulfide Isomerase (PDI), Inositol-Requiring Enzyme1α (IREα), and Immunoglobulin Heavy Chain Binding Protein (Bip). The main function of these proteins is to reduce the endoplasmic reticulum load and proteins unfolding and misfolding (10). Therefore, it is of great significance to study the effect of Apoptin on the expression of ER and ER stress related proteins to explore the regulatory function of ER metabolism.

ER functional structure damage can significantly affect protein, lipid and carbohydrate synthesis, resulting in serious impacts on tumor cells’ proliferation and metastasis. As one of the main components of the tumor cell membrane, lipids are closely related to the migration and invasion of tumor cells (11). However, the mechanism by which lipid metabolism affects tumor cell migration and invasion is unclear. The most important enzymes in lipid metabolism, such as Acetyl-CoA Carboxylase (ACC), Fatty Acid Synthase (FASN), ATP Citrate Lyase (ACLY), Phospholipase D1 (PLD1), Stearoyl-CoA Desaturases 1 (SCD1), are closely related to cancer metastasis (11). Meanwhile, there are no reports about the effects Apoptin-induced ER stress injury on lipid metabolism, migration and invasion of HepG-2 cells. Therefore, it is also important to study the relationship between lipid metabolism and migration and invasion.

In this study, we show that Apoptin can cause a strong and lasting ER stress response that damages ER functional structure, and significantly affects the lipid metabolism level and the migration and invasion abilities of HepG-2 cells. For this, we used methods that assessed changes in endoplasmic reticulum, lipid metabolism, migration, and invasion, using an immunohistochemistry approach of tissue from tumor-bearing nude mouse model.

## Materials and Methods

### Materials

The human liver cancer cell line HepG-2 was purchased from the Cell Bank of the Chinese Academy of Sciences (Shanghai, China). We used a recombinant type 5 adenovirus Ad5-CMV-Apoptin (Ad-Vp3) that contained a CMV promoter and the chicken anemia virus Apoptin gene. The recombinant type 5 adenovirus Ad5-CMV (Ad-Mock) without Apoptin gene was used as a blank control. Ad-Vp3 and Ad-Mock were previously constructed in our laboratory.

Six-week-old male BALB/c nude mice were purchased from the Experimental Animal Center of The Academy of Military Medical Sciences of China. The animal experimental protocols were approved by the Institutional Animal Care and Use Committee (IACUC) of the Chinese Academy of Military Medical Science, Changchun, China (10ZDGG007). All surgeries were performed under sodium pentobarbital anesthesia and all efforts were made to minimize suffering.

The Cell Counting Kit-8 (No.CD04) was purchased from DOjinDO (Shanghai, China). The human LDH ELISA Kit (No.69-98628), human Reactive Oxygen Species (ROS) ELISA Kit (No.69-99364), human TC ELISA Kit (No.69-52377), and human TG ELISA Kit (No.69-99502) were purchased from MSK (Wuhan, China). Hoechst 33342 (No.H1399), JC-1 (No.T3168), ER-Tracker™ Green (No.E34251) were purchased from ThermoFisher Scientific (Shanghai, China). The FITC Annexin V Apoptosis Detection Kit I (No.556547) was purchased from BD Biosciences (Beijing, China). The Minute™ Total Protein Extraction Kit (No.SD-001/SN-002) was purchased from invent. The ER Stress Antibody Sampler Kit (No.9956), Fatty Acid and Lipid Metabolism Antibody Sampler Kit (No.8335), SCD1 (C12H5) Antibody (No.2794), PLD1 Antibody (No.3832), Phospho-PLD1 (Thr147) Antibody (No.3831), Girdin Antibody (No.14200S), Palladin (D9H2) Rabbit mAb (No.8518S), E-Cadherin (24E10) Rabbit mAb (No.3195S), N-Cadherin (D4R1H) XP^®^ Rabbit mAb (No.13116S), Vimentin (D21H3) XP^®^ Rabbit mAb (No.5741S) were purchased from CST. The Transwell^®^ Permeable Supports 6.5 mm Insert, 24 Well Plate 8.0 μm Polycarbonate Membrane (No.3422), BioCoat Corning^®^ Matrigel^®^ Invasion Chamber 24-well plate 8.0 Micro (No.354480) and Matrigel^®^ Matrix Basement Membrane (No:356234) were purchased from CORNING. The 25 Culture-Inserts 2 well for self-insertion (No.80209) was purchased from ibidi.

### Methods

#### Cell Viability Analysis

The HepG-2 human hepatoma cells were seeded at 5 × 10^3^ cells per well in a 96-well plate and incubated for 24h at 37°C with 5% CO_2_. HepG-2 cells were infected with Ad-Vp3 at 10 multiplicity of infection (MOI), 50 MOI and 100 MOI. Along with Ad-Mock-infected control wells, the infected cells were cultured at 37°C with 5% CO_2_ for 24, 48, and 72 h. Cell viability was measured by the Cell Counting Kit-8 and Human LDH ELISA Kit, following the manufacturer’s instructions. Ad-Mock-infected cells and HepG-2 cells were used as negative controls.

#### Hoechst Staining

HepG-2 cells were seeded at 6 × 10^5^ cells/well into a 6-well plate, cultured at 37°C with 5% CO_2_ for 24 h and infected with Ad-Vp3 at 10 MOI, 50 MOI, and 100 MOI for 24, 48, and 72 h. The infected cells were stained with 1,000-fold dilutions of Hoechst and incubated at 37°C away from light for 5 min and then the karyomorphism of infected cells were observed through a fluorescence microscope. Ad-Mock-infected cells and HepG-2 cells were used as negative controls. The results of cell viability analysis and Hoechst staining were determined based on cells’ infection with Ad-Vp3 as 50 MOI.

#### Annexin V Analysis

HepG-2 cells were seeded at 6 × 10^5^ cells per well in a 6-well plate and incubated for 24h at 37°C with 5% CO_2_. The cells were then infected with 50 MOI Ad-Vp3 for 24, 48, and 72 h. The Ad-Vp3-infected HepG-2 cells were incubated in the dark for 15 min at room temperature in the presence of 5 μl Annexin V-FITC and 5μl PI in 100 μl of 1 × binding buffer, and then the apoptosis of HepG-2 cells was analyzed by a fluorescence microscope and flow cytometry. Ad-Mock -infected cells and HepG-2 cells were used as negative controls.

#### ROS Levels Elisa Analysis

HepG-2 cells were seeded at 6 × 10^6^ cells per well in a 6-well plate and incubated for 24 h at 37°C with 5% CO_2_. The cells were then infected with 50 MOI Ad-Vp3 for 24, 48 and 72 h. The ROS levels of Ad-Vp3-infected HepG-2 cells were detected by a human ROS ELISA Kit and following the manufacturer’s instructions. Ad-Mock -infected cells and HepG-2 cells were used as negative controls.

#### Mitochondrial Membrane Potential Analysis

HepG-2 cells were seeded at 6 × 10^5^ cells/well into a 6-well plate, cultured for 24 h at 37°C with 5% CO_2_ and infected with Ad-Vp3 at 50 MOI for 12, 24, 36, 48, and 72 h. The infected cells were incubated in the dark for 20 min at 37°C in the presence of 1 μL JC-1 in 1 ml of DMEM, and then the mitochondrial membrane potential of HepG-2 cells was analyzed by a fluorescence microscope and flow cytometry, following the manufacturer’s instructions. Ad-Mock-infected cells and HepG-2 cells were used as negative controls.

#### Endoplasmic Reticulum Flow Cytometry and Fluorescence Staining Analysis

HepG-2 cells were seeded at 6 × 10^5^ cells/well into a 6-well plate, cultured for 24 h at 37°C with 5% CO_2_ and infected with Ad-Vp3 at 50 MOI for 12, 24, 36, 48, and 72 h. The infected cells were incubated in the dark for 30 min at 37°C, in the presence of 4 μM ER-Tracker™ Green, and then the Endoplasmic reticulum of HepG-2 cells was analyzed by a fluorescence microscope and flow cytometry, following the manufacturer’s instructions. Ad-Mock-infected cells and HepG-2 cells were used as negative controls.

#### Western Blotting

HepG-2 cells were seeded at 6 × 10^5^ cells/well into a 6-well plate, cultured for 24 h at 37°C with 5% CO_2_ and infected with Ad-Vp3 at 50 MOI for 12, 24, 36, 48, and 72 h. The total protein of the infected cells was extracted by Minute™ Total Protein Extraction Kit and probed for expression of ER stress related proteins and lipid metabolism related proteins by Western-blot, following the manufacturer’s instructions. Ad-Mock-infected cells were used as negative controls.

#### TG and TC Level Elisa Analysis

HepG-2 cells were seeded at 6 × 10^5^ cells/well into a 6-well plate, cultured for 24 h at 37°C with 5% CO_2_ and infected with Ad-Vp3 at 50 MOI for 12, 24, 36, 48, and 72 h. The levels of TG and TC in infected cells were detected by the human TC ELISA Kit and human TG ELISA Kit, following the manufacturer’s instructions. Ad-Mock-infected cells and HepG-2 cells were used as negative controls.

#### Migration and Invasion Detection

HepG-2 cells were seeded at 1 × 10^4^ cells per well in a 24-well plate and cultured for 24 h at 37°C with 5% CO_2_. The cells were infected with Ad-Vp3 at 50 MOI for 24 and 48 h. After trypsinization, the HepG-2 cells were seeded in the upper chamber of the cell culture inserts and cultured for 24 h. The cells that migrated through the membrane were counted under a microscope and after they were fixated by carbinol and stained with crystal violet. The experimental procedure of matrigel invasion assay was similar to the transwell migration assay except for incubation with matrigel on the upper chamber for 1 h and before cells’ seeding. Ad-Mock-infected cells and HepG-2 cells were used as negative controls.

#### Scratch Test

HepG-2 cells were seeded at 7 × 10^5^ cells/well into a 25 Culture-Inserts 2 well for self-insertion, cultured for 24 h at 37°C with 5% CO_2_ and then the Culture-Inserts were taken down. The cells were infected with Ad-Vp3 at 50 MOI for 0, 24, 48, and 72 h. Scratches were performed, and their widths were measured by ImageJ (version 1.51j8; National Institutes of Health, USA) at the indicated time points and according to the formula: migration rate = (0 h scratch widths – 24 h or 48 h or 72 h scratch widths)/(0 h scratch widths. Ad-Mock-infected cells and HepG-2 cells were used as negative controls.

#### *In Vivo* Studies

The xenograft models were established *via* subcutaneous injection of HepG-2 cells (5 × 10^6^/100 μl) with Matrigel^®^ Matrix Basement Membrane (yielding a 1:1 ratio) into mice right legs. When the tumors were clearly observable (usually at 14 days), the mice were divided randomly into three groups (n = 39): group 1 was infected with 1 × 10^8^ plaque forming units (PFU) of Ad-VT in 100 μl of PBS, group 2 was infected with 1 × 10^8^ PFU of Ad-Mock in 100 μl of PBS and group 3 was injected with 100μl of PBS. All groups were treated *via* intratumoral injection from day 14. The injections were given every 3 days for 15 days. The xenograft tumors’ length and width were measured and observed every 7 days for 42 days using Vernier calipers. The survival condition of the tumor-bearing nude mice was recorded every 7 days for 56 days. During the experiment, tumors from three mice and from each group were harvested. We selected the ER stress, lipid metabolism, migration, and invasion related proteins, such as PERK, Calnexin, Ero1-Lα, BIP, ACC, p-ACC, FASN, PLD1, p-PLD1, SCD1, Palladin, N-Cadherin, E-Cadherin, and Vimentin, for *in vitro* assessment using immunohistochemistry staining. We commissioned the Servicebio to complete the immunohistochemical detection.

#### Statistics

The statistical analyses were performed using the Statistical Package for the Social Sciences (SPSS) statistical software package (version 15.0; SPSS Inc., Chicago, IL, USA), and the results were obtained using GraphPad Prism version 7.0 (GraphPad Software Inc., La Jolla, CA, USA). Student’s t-test or one-way analysis of variance followed by Tukey’s *post hoc* test were used. Differences with a p < 0.05 or p < 0.01 or P < 0.001 were considered statistically significant. Data are presented as the mean ± standard deviation (SD) values.

The immunohistochemical analysis was performed using the Image-Pro Plus 6.0 (Media Cybernetics, Inc., Rockville, MD, USA). At least three fields of vision were randomly selected for each slice and in each group. When taking photos, we made a full organization of the whole field of vision to ensure that the background light of each photo was consistent. The analytical method of KI67 and Tunel was based on the presence of a brownish yellow staining of the nucleus and that was selected as the uniform standard to judge the positive cells in all photos, and a blue nucleus was selected for the other cells. The number of positive cells in each photo and the total number of cells were obtained using Image-Pro Plus 6.0. Positive rate = Positive cells number/Total cell count × 100%. The analytical method used to assess the expression of other proteins was also based on the presence of brownish yellow staining was selected as a unified standard to judge positivity. The accumulated optical density (IOD) and the area of the tissue (AREA) were obtained using Image-Pro Plus 6.0. Areal density = IOD/AREA. A larger areal density value indicated a higher positive expression level.

## Results

### Inhibition of HepG-2 Cells’ Proliferation Inhibited by Apoptin

**Figure 1:**
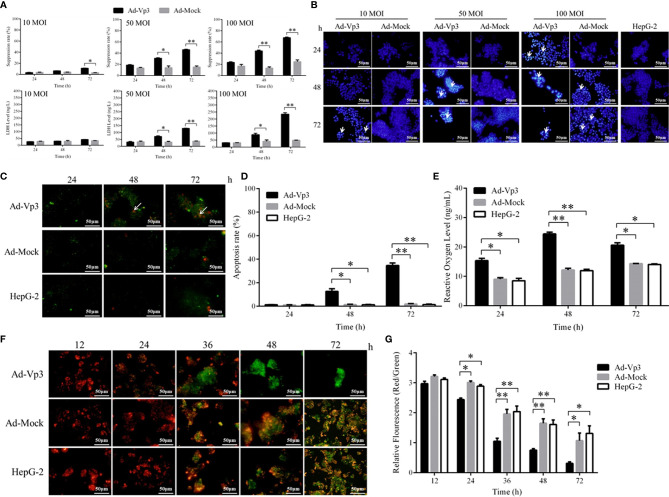
Inhibitory effect of Apoptin on HepG-2 cells *in vitro*. Apoptin can significantly induce apoptosis, increase activity level and decrease mitochondrial membrane potential of Ad-Vp3-infected HepG-2 cells. **(A)** The results of inhibitory effect in Ad-Vp3-infected HepG-2 cells detected by Cell Counting Kit-8 and LDH ELISA Kit. **(B)** Hoechst staining (200×). **(C)** Annexin V-FITC/PI staining (200×). **(D)** Annexin V-FITC/PI flow cytometry detection. **(E)** Intracellular reactive oxygen species detection by ELISA. **(F)** JC-1 staining (200×). **(G)** JC-1 relative fluorescence detection. Data are presented as mean ± SD, *p < 0.05, **p < 0.01.

The proliferation of HepG-2 cells can be significantly suppressed by Apoptin (**Figure 1A**). There was no significant difference in growth suppression between Ad-Vp3 and Ad-Mock infected cells at MOI of 10 (p > 0.05). While at MOI of 50 or 100, the growth suppression of Ad-Vp3-infected cells was significantly higher than that in Ad-Mock-infected cells 48 and 72 h post-infection (p < 0.05), however the difference was not significant at 24 h. Nuclear fragmentation assessed by Hoechst staining, was readily observed in Ad-Vp3-infected cells (**Figure 1B**). While at MOI of 100, Ad-Mock had some inhibitory effects on inhibiting HepG-2 cells. Therefore, we determined Ad-Vp3 optimal MOI as 50 MOI. HepG-2 cells’ apoptosis can be significantly induced by Apoptin (**Figures 1C, D** **)**. Apoptosis assessed by Annexin V-FITC/PI staining, was readily observed in Ad-Vp3-infected cells at 48 and 72 h post-infection. The apoptotic rate in Ad-Vp3-infected cells was significantly higher than that in Ad-Mock-infected cells and HepG-2 cells at 48 and 72 h post-infection (p < 0.05). HepG-2 cells’ ROS level can be significantly induced by Apoptin (**Figure 1E**). ROS level in Ad-Vp3-infected cells was significantly higher than that in Ad-Mock-infected cells and HepG-2 cells 24, 48, and 72 h post-infection (p < 0.05). HepG-2 cells’ mitochondrial membrane potential can be significantly reduced by Apoptin (**Figures 1F, G** **)**. Following JC-1 staining, fluorescence in HepG-2 cells rapidly turned from red to green 24 h and 72 h after infection with Ad-Vp3 at MOI of 50. The relative fluorescence (Red/Green) of Ad-Vp3-infected cells was significantly lower than that of Ad-Mock-infected cells and HepG-2 cells 24, 36, 48, and 72 h post-infection (p < 0.05). These results showed that HepG-2 cells’ growth, ROS level and mitochondrial membrane potential can be significantly suppressed by Apoptin *in vitro*.

### Apoptin Induction of Endoplasmic Reticulum Stress

**Figure 2:**
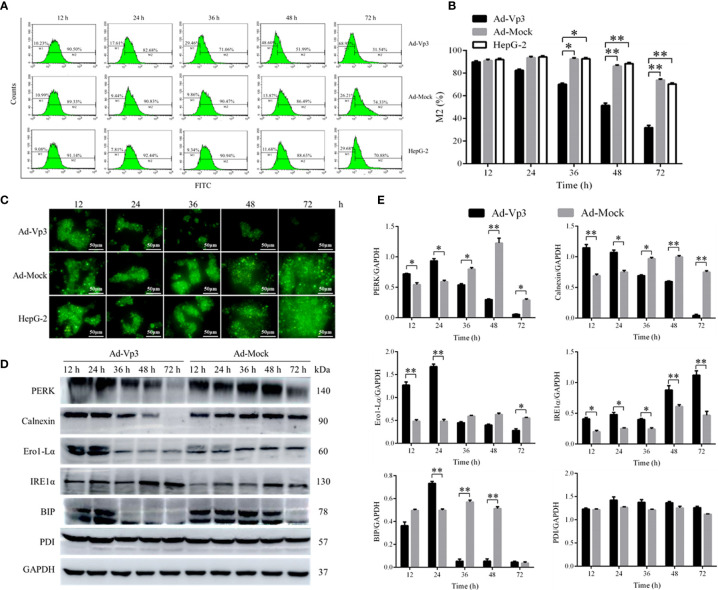
Endoplasmic reticulum related detection. The endoplasmic reticulum stress of Ad-Vp3-infected HepG-2 cells can be stimulated by Apoptin, 24 h and 48 h post-infection, and ER functional structure is gradually impaired gradually from 36 h to 72 h post-infection. **(A, B**) Flow cytometry detection of ER using the ER-Tracker™ Green. **(C)** Endoplasmic reticulum ER-Tracker™ Green staining (200×). **(D, E)** Western-blot detection of endoplasmic reticulum stress related proteins. Data are presented as mean ± SD, *p < 0.05, **p < 0.01.

The effect of Apoptin on the ER functional structure of HepG-2 cells was evaluated using flow cytometry and fluorescence. The Green fluorescence intensity of endoplasmic reticulum in Ad-Vp3-infected cells was significantly lower than that in Ad-Mock-infected cells and HepG-2 cells 36, 48 and 72 h post-infection (**Figures 2A, B** **)** (p < 0.05). The variation trend of the green fluorescence intensity was similar to that shown by flow cytometry (**Figure 2C**). The expression of ER related proteins can be significantly affected by Apoptin **(** **Figures 2D, E** **)**. The expression of PERK, Calnexin, Ero1-Lα and BIP in Ad-Vp3-infected cells were significantly higher at 12 and 24 h, and significantly lower at 36, 48, and 72 h post-infection when compared with the Ad-Mock infected cells (p < 0.05). The expression of IRE1α in Ad-Vp3-infected cells was significantly higher than that in Ad-Mock-infected cells at and at all-time points (p < 0.05). There were no significant differences in the expression of PDI in HepG-2 cells infected with Ad-Vp3 and Ad-Mock (p > 0.05). These results indicate that the partial expression of ER related proteins can be increased by ER stress, and some of these proteins can be reduced by ER stress injury. Therefore, ER stress in HepG-2 cells can be induced by 50 MOI Ad-Vp3, 12 and 24 h post-infection. ER functional structure can be damaged by a strong and lasting ER stress, which was induced by 50 MOI Ad-Vp3, 36, 48, and 72 h post-infection.

### Effect of Apoptin on Lipid Metabolism

**Figure 3:**
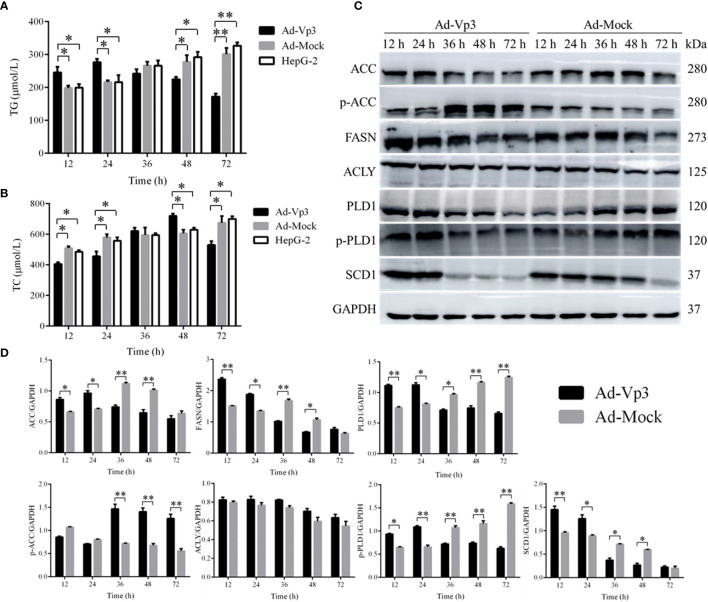
Lipid metabolism related detection. Detection of Lipid metabolism and migration rates. Apoptin can significantly change TG and TC levels, affect expression of lipid metabolism related enzymes, and reduce migration rate in Ad-Vp3-infected HepG-2 cells. **(A)** TG level Elisa detection. **(B)** TC level detection by Elisa. **(C, D)** Western blot analysis of the expression levels of enzymes closely related to migration and invasion in lipid metabolism. Data are presented as mean ± SD, *p < 0.05, **p < 0.01.

The lipid metabolism of HepG-2 cells can be significantly affected by Apoptin. TG level in Ad-Vp3-infected cells was significantly higher than that in Ad-Mock-infected cells and HepG-2 cells, 12 and 24 h post-infection (p < 0.05), while that in Ad-Vp3-infected cells was significantly lower than that in Ad-Mock-infected cells and HepG-2 cells, 48 and 72 h post-infection (**Figure 3A**) (p < 0.05). TC level in Ad-Vp3-infected cells was significantly lower than in Ad-Mock-infected cells and HepG-2 cells, 12 and 24 h post-infection (p < 0.05), while that in Ad-Vp3-infected cells was significantly higher than in Ad-Mock-infected cells and HepG-2 cells, 36, 48 and 72 h post-infection (**Figure 3B**) (p < 0.05). The expression of lipid metabolism related enzymes can be significantly affected by Apoptin (**Figures 3C, D** **)**. The expression of lipid metabolism related enzymes ACC, FASN, PLD1, p-PLD1, and SCD1 in Ad-Vp3-infected cells were significantly higher at 12 and 24 h, and significantly lower at 36, 48, and 72 h post-infection when compared with the Ad-Mock infected cells (p < 0.05). The variation trend of p-ACC expression is opposite to that of ACC, due to ACC inactivation that could be caused by phosphorylation. There was no significant difference in ACLY expression between Ad-Vp3-infected and Ad-Mock-infected cells at various time points (p > 0.05).

### Apoptin Reduced the Migration and Invasion Abilities of HepG-2 Cells

**Figure 4:**
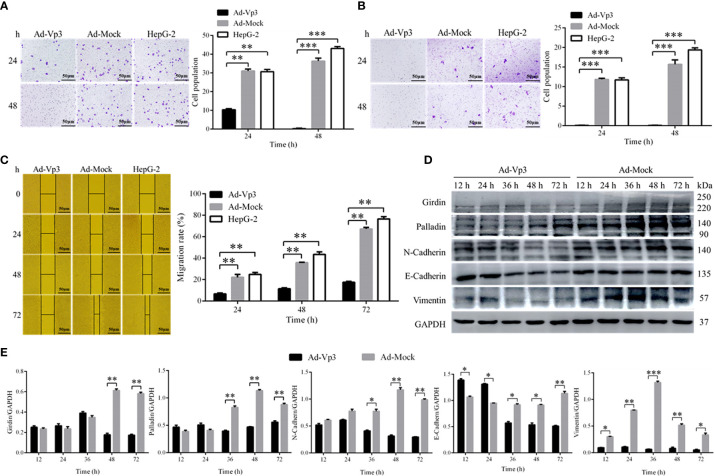
Migration, invasion, and scratch test related detection. Analysis of Migration, invasion, and scratch test. Apoptin can significantly reduce migration and invasion, affect the expression of migration and invasion related proteins. **(A)** The results of migration test (100×). **(B)** The results of invasion test (100×). **(C)** The results of scratch assay (100×). **(D, E)** Western blot analysis of the expression of migration and invasion related proteins. Data are presented as mean ± SD, *p < 0.05, **p < 0.01, ***p < 0.001.

The migration and invasion of HepG-2 cells can be significantly reduced by Apoptin. The number of migrating cells in HepG-2 cells infected with Ad-Vp3 for 24 and 48 h, was significantly lower than that in the other groups (**Figure 4A**) (p < 0.01). The number of invading cells in HepG-2 cells infected with Ad-Vp3 for 24 and 48 h, was significantly lower than that in the other groups (**Figure 4B**) (p < 0.01). The migration rate of HepG-2 cells can be significantly suppressed by Apoptin (**Figure 4C**) (p < 0.01). The migration rate suppression in Ad-Vp3-infected cells was significantly lower than that in Ad-Mock-infected cells and HepG-2 cells, 24, 48, and 72 h post-infection (p < 0.05). These results showed that lipid metabolism and migration rate can be significantly affected by Apoptin. Furthermore, the expression of migration and invasion related proteins Girdin, Palladin, Vimentin, N-Cadherin, and E-Cadherin in HepG-2 cells can be significantly suppressed by Apoptin expression (**Figures 4D, E** **)**. The expression levels of Girdin, Palladin, and N-Cadherin, in Ad-Vp3-infected cells were significantly lower than those in Ad-Mock-infected cells at 48 and 72 h (p < 0.05). The expression level of E-Cadherin in Ad-Vp3-infected cells was significantly higher at 12 and 24 h than that in cells infected with Ad-Mock, but were significantly lower at 36 h, 48 h, and 72 h post-infection (p < 0.05). The expression level of Vimentin in Ad-Vp3-infected cells was significantly lower than that in Ad-Mock-infected cells and at all-time points (p < 0.05). These results showed that Apoptin can significantly reduce migration and invasion of HepG-2 cells, as also reflected by the decrease in the expression of migration and invasion related proteins.

### Apoptin Inhibited the Proliferation of HepG-2 Cells *In Vivo*

**Figure 5:**
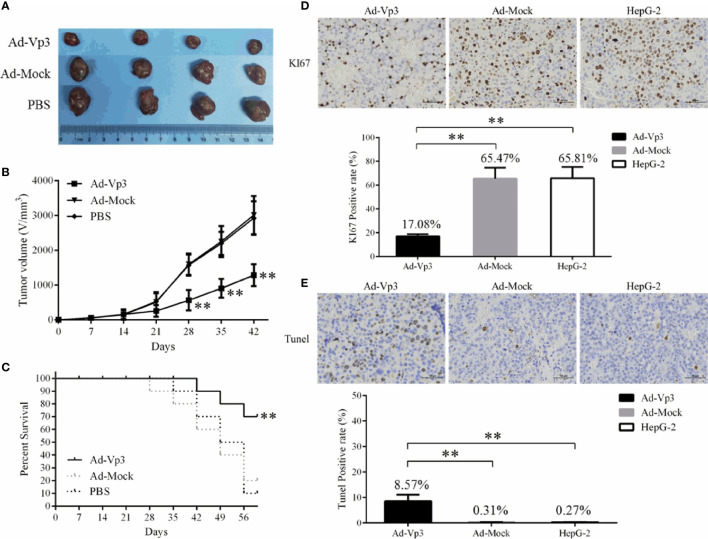
Inhibitory effect of Apoptin on HepG-2 cells *in vivo*. Apoptin significantly inhibits tumor growth and prolongs survival of tumor bearing nude mice. **(A, B)** The result of tumor growth inhibition. **(C)** The results of survival rate. **(D, E)** The results of immunocytochemistry detection of KI67 and Tunel in tumors’ tissue (400×). Data are presented as mean ± SD, **p < 0.01.

The proliferation of HepG-2 cells can be significantly suppressed by Apoptin in tumor bearing nude mice. The tumor volume in the Ad-Vp3 group was significantly lower than that in the Ad-Mock group and HepG-2 group, 28 to 42 days after tumor xenografting. (**Figures 5A, B** **)** (p < 0.01). The survival rate of the Ad-Vp3 group was 70%, while that of the Ad-Mock and HepG-2 groups were 20% and 10%, 56 days after tumor xenografting, respectively. The survival rate in the Ad-Vp3 group was significantly longer than that in the Ad-Mock and HepG-2 groups, 56 days after tumor xenografting (**Figure 5C**) (p < 0.01). The KI67 positivity rate in the Ad-Vp3 group tumors was 17.08%, while that of the Ad-Mock and HepG-2 groups were 65.47% and 65.81%, respectively. The KI67 positivity rate in the Ad-Vp3 group tumors was significantly lower than that in the Ad-Mock and HepG-2 groups (**Figure 5D**) (p < 0.01). The Tunel positivity rate in the Ad-Vp3 group tumors was 8.57%, while that of the Ad-Mock and HepG-2 groups were 0.31% and 0.27%, respectively. The Tunel positivity rate in the Ad-Vp3 group tumors was significantly higher than that in the Ad-Mock and HepG-2 groups (**Figure 5E**) (p < 0.01). These results showed that Apoptin could significantly inhibit HepG-2 cell proliferation *in vivo*.

### Immunohistochemical Detection of ER Stress, Lipid Metabolism, and Invasion Related Proteins in Tumor Tissues

**Figure 6:**
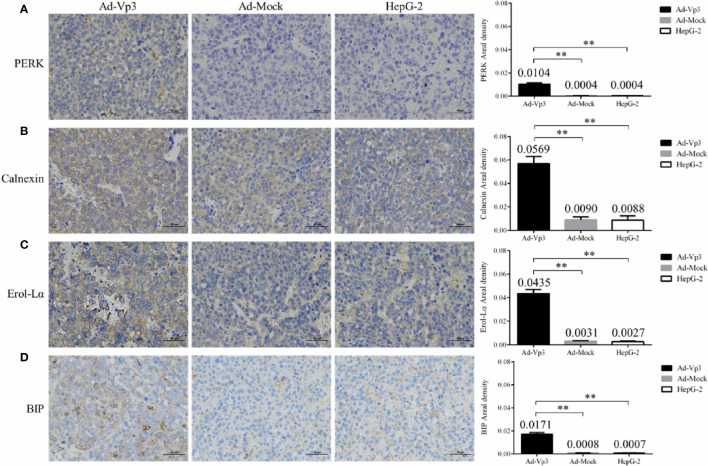
Immunohistochemical detection of endoplasmic reticulum stress related proteins in tumor tissues. **(A–D)** The expression of endoplasmic reticulum stress related proteins (PERK, Calnexin, Ero1-Lα, BIP) in tumors’ tissue were detected by immunocytochemistry (400×). Data are presented as mean ± SD, **p < 0.01.

**Figure 7:**
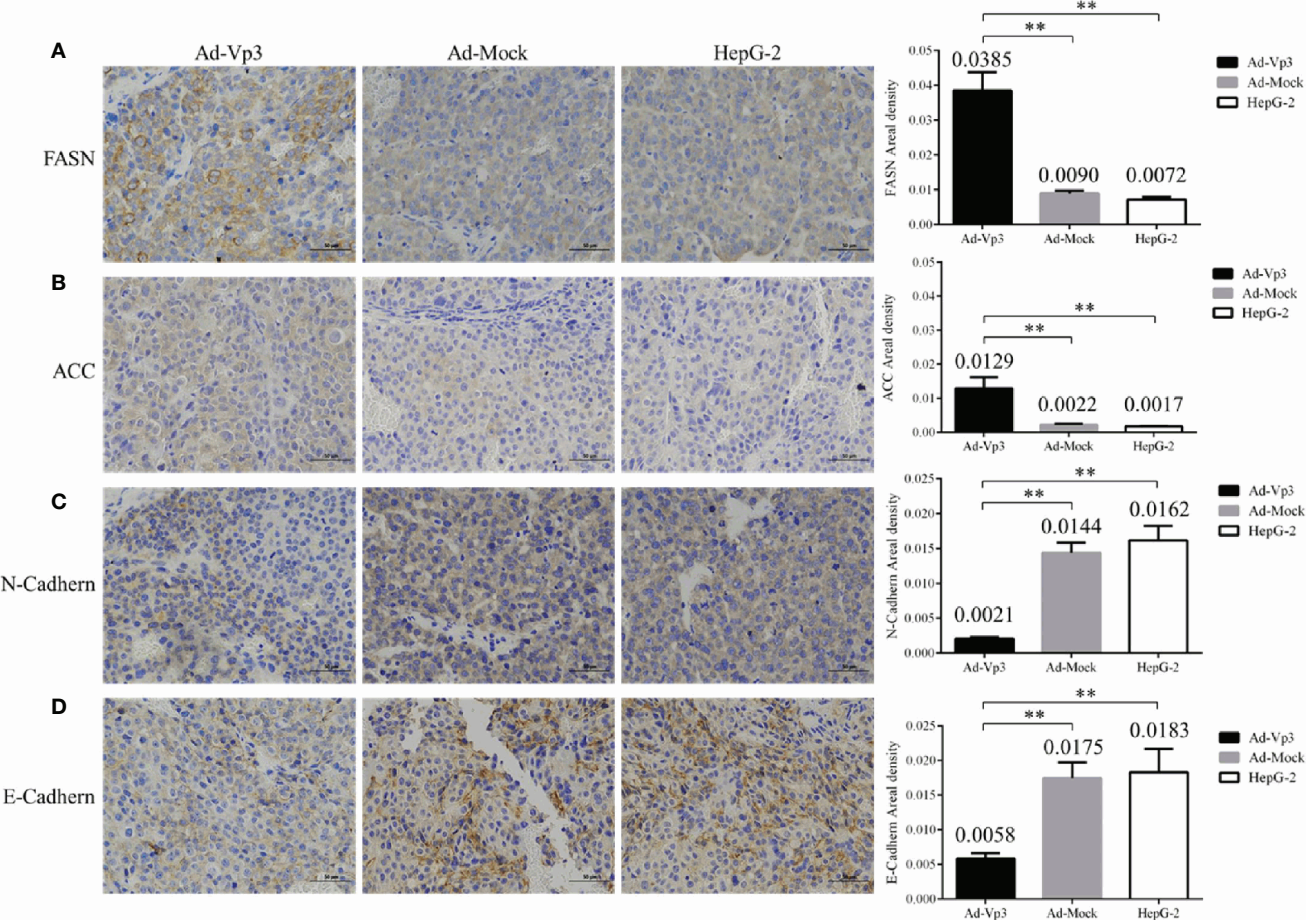
Immunohistochemical detection of lipid metabolism and invasion related proteins in tumor tissues. **(A–D)** The expression of lipid metabolism related proteins (FASN, ACC) and invasion related proteins (N-Cadherin, E-Cadherin) were detected by immunocytochemistry (400×). Data are presented as mean ± SD, **p < 0.01.

The ER stress, lipid metabolism and invasion related protein can be significantly affected by Apoptin *in vivo*. These changes can be assessed using immunohistochemistry (IHC). The immunohistochemical detection results of ER stress proteins are shown in **Figures 6A–D**. The areal densities of PERK, Calnexin, Ero1-Lα, and BIP in the Ad-Vp3 group tumors were significantly higher than those in the Ad-Mock and HepG-2 groups (p < 0.01). The IHC results of lipid metabolism related proteins are shown in **Figures 7A, B**. The areal densities of FASN and ACC in the Ad-Vp3 group tumors were significantly higher than those in the Ad-Mock and HepG-2 groups (p < 0.01). IHC results of invasion related proteins are shown in **Figures 7C, D**. The areal densities of N-Cadherin and E-Cadherin in the Ad-Vp3 group tumors were significantly higher than those in the Ad-Mock and HepG-2 groups (p < 0.01). These IHC results showed that Apoptin could significantly induce the expression of partial ER stress and lipid metabolism related proteins, and significantly reduce the expression of partial invasion related proteins *in vivo*. There were no significant differences in the expression of the others endoplasmic reticulum stress, lipid metabolism, migration, and invasion related proteins, such as p-ACC, PLD1, p-PLD1, SCD1, Palladin, and Vimentin, for each group. Therefore, these results have been omitted.

## Discussion

ER stress is a physiological and pathological process in which the function of ER is disturbed due to various reasons. During this process, the body can upregulate the expression of ER chaperones, inhibit protein translation, start the degradation of ER related proteins, improve the physiological state of cells and strengthen the self-repair function of endoplasmic reticulum through increasing the expression of the stress related proteins PERK, Calnexin, Ero1-Lα, PDI, IRE1α, and BIP. PERK (12–14) is a transmembrane protein of the eIF2α kinase that is located in ER, and that couples the ER stress signal and inhibit translation. Calnexin (15–17) is an ER calcium binding protein that assists in protein folding and participates in quality control. Ero1-Lα (18–20) is a key molecule that mediates PDI (21, 22) catalyzed protein oxidative folding to form disulfide bonds. They work together to provide disulfide bonds for newly synthesized proteins to ensure the formation of natural conformations. IRE1α (23–25) is an endoplasmic type I transmembrane protein with a serine/threonine kinase and ribonucleic acid endonuclease activities, that participates in cell apoptosis, induced by unfolded protein response and endoplasmic reticulum stress response. The main function of BiP (26–28) is to assist the correct folding of newly synthesized proteins. In this study, we found that the expression of the ER stress related proteins PERK, Calnexin, Ero1-l α, BIP, and PDI was significantly higher in the Ad-Vp3-infected HepG-2 cells compared to Ad-Mock-infected HepG-2 cells, 12 h and 24 h post-infection. These decreases may be caused by ER stress. However, the expression of these proteins in Ad-Vp3-infected cells were significantly lower than those in the Ad-Mock-infected cells and the intensity of the green fluorescence also decreased significantly, 36 h, 48 h, and 72 h post-infection. By combining these two results, we speculated that the ER function and structure may be damaged by the strong and persistent ER stress, resulting in an increased expression of ER related proteins, followed by their decrease. In conclusion, we suggest that Apoptin can damage the ER function in HepG-2 cells by causing a strong and persistent ER stress.

The strong and lasting ER stress can seriously damage ER functional structure and affect the cells’ normal lipid metabolism (29). The enzymes involved in lipid metabolism mainly include FANS, ACC, ACLY, PLD, and SCD1. FASN (30, 31) is a kind of multifunctional polypeptide enzyme which can produce fatty acids. It is the catalyst for the last step of fatty acid synthesis. ACC (32, 33) is the rate-limiting enzyme in fatty acid synthesis. ACLY (34, 35) is a key enzyme that converts citric acid into oxaloacetate and fatty acyl-CoA, responsible for the synthesis of new fatty acids. Phosphatidylcholine (PC) and Phosphatidyl Ethanolamine (PE), the hydrolysates of PLD (36), are the main components of membrane lipids and the key molecules of tumor cell invasion and metastasis (37, 38). Several saturated fatty acyl-CoAs can be catalyzed by SCD (39, 40), that mainly produces palmitoleic acid and oleic acid, which provide key substrates for complex lipids production, such as phospholipids, triglycerides and cholesterol esters. It has been reported that lipid synthesis and metabolism can be affected by ER stress (41–43). Among the players involved, the PERK pathway is involved in the expression of lipid metabolism related genes, and PERK functional loss can reduce the expression of the fatty acid synthesis related genes FASN, ACL, and SCD1 (44). In this study, we found that the changing trend in FANS, ACC, PLD1, and SCD1 expression was basically the same as that of the ER stress related proteins. The expression of these enzymes in the Ad-Vp3-infected cells were significantly higher than those in the Ad-Mock-infected HepG-2 cells, 12 h and 24 h post-infection, while the expression of these enzymes in the Ad-Vp3-infected cells were significantly lower than those in the Ad-Mock-infected HepG-2 cells, 36 h, 48 h, and 72 h post-infection. Therefore, we speculate that the expression of lipid metabolism related enzymes may be affected by the ER stress damage.

The results of migration and invasion experiments showed that Apoptin can affect the expression of proteins involved in HepG-2 migration and invasion and therefore, reduce their abilities to migrate and invade. Some enzymes involved in lipid metabolism are closely related to tumor cell migration and invasion. FASN participates in the synthesis of phospholipids required for cell membranes’ construction. Meanwhile, FASN main product is palmitic acid, which is the main component of cell membrane decoupling. FASN overexpression is closely related to the degree of malignant tumor progression (45–47). ACC high expression is also significantly related to the characteristics of multiple aggressive clinical liver cancer cases, such as vascular infiltration and poor differentiation) (48). However, this activity is inhibited by ACC phosphorylation (p-ACC), which inactivates ACC (49). SCD1 is located in the ER and is involved in lipid biosynthesis and invasion (50). PLD1 induced expression through the RAF/ERK and NF-κB signaling pathways, leads to MMP-9 increased secretion (51), which promotes tumor invasion and metastasis. There is a definite relation between these lipid metabolism related enzymes and tumor cell migration and invasion. Therefore, we preliminarily speculated that the abnormal expression of these enzymes during lipid metabolism may be the one of the main inducements that affect HepG-2 migration and invasion.

The liver is rich in ER and mitochondria and has a high lipid metabolism. Therefore, to explore the effect of endoplasmic reticulum stress, induced by Apoptin on lipid metabolism, migration and invasion, we selected HepG-2 as a cellular model to study the relationship between the 3 above processes. To further confirm the role of Apoptin in the induction of ER stress, we also studied Apoptin involvement in the other hepatoma cell lines SMMC-7721 and QGY-7703. However, we did not obtain the expected results. Western-blot detection results of endoplasmic reticulum stress related proteins showed that the endoplasmic reticulum stress response in SMMC-7721 and QGY-7703 cells, infected by 50 MOI Ad-Vp3, were not significantly different. In the next step, we may choose to use other hepatoma cells or other types of tumor cells to verify the universality of Apoptin-induced ER stress. We will also further explore the effect of Apoptin induced endoplasmic reticulum stress damage on the apoptotic pathway and Ca2 + level in HepG-2 cells’ ER. We need also to further explore the effects of Apoptin-induced ER stress injury on the ER apoptotic pathway and Ca^2+^ level in HepG-2 cells.

## Data Availability Statement

The original contributions presented in the study are included in the article/supplementary material. Further inquiries can be directed to the corresponding authors.

## Ethics Statement

The animal study was reviewed and approved by Institutional Animal Care and Use Committee (IACUC) of the Chinese Academy of Military Medical Science, Changchun, China (10ZDGG007).

## Author Contributions

NJ, GZ, and XL designed this study. YZ, YL, CS, JC, WL, SL, GS, and ZL performed experiments. YZ, BB, and JZ analyzed data. YZ and BB wrote the manuscript. JF revised the manuscript. All authors contributed to the article and approved the submitted version.

## Conflict of Interest

The authors declare that the research was conducted in the absence of any commercial or financial relationships that could be construed as a potential conflict of interest.

## References

[1] Jeurissen SH Wagenaar F Pol JM van der Eb AJ Noteborn MH. Chicken anemia virus causes apoptosis of thymocytes after in vivo infection and of cell lines after in vitro infection. J Virol (1992) 66(12):7383–8. 10.1128/JVI.66.12.7383-7388.1992 PMC240444 1331529

[2] Bassermann F Frescas D Guardavaccaro D Busino L Peschiaroli A Pagano M. The Cdc14B-Cdh1-Plk1 axis controls the G2 DNA-damage-response checkpoint. Cell (2008) 134(2):256–67. 10.1016/j.cell.2008.05.043 PMC2591934 18662541

[3] Heilman DW Teodoro JG Green MR. Apoptin nucleocytoplasmic shuttling is required for cell type-specific localization, apoptosis, and recruitment of the anaphase-promoting complex/cyclosome to PML bodies. J Virol (2006) 80(15):7535–45. 10.1128/JVI.02741-05 PMC1563728 16840333

[4] Teodoro JG Heilman DW Parker AE Green MR. The viral protein Apoptin associates with the anaphase-promoting complex to induce G2/M arrest and apoptosis in the absence of p53. Genes Dev (2004) 18(16):1952–7. 10.1101/gad.1198404 PMC514174 15314021

[5] Chaabane W Ghavami S Malecki A Los MJ. Human Gyrovirus-Apoptin Interferes with the Cell Cycle and Induces G2/M Arrest Prior to Apoptosis. Arch Immunol Ther Exp (Warsz) (2017) 65(6):545–52. 10.1007/s00005-017-0464-8 28386695

[6] Maddika S Booy EP Johar D Gibson SB Ghavami S Los M. Cancer-specific toxicity of apoptin is independent of death receptors but involves the loss of mitochondrial membrane potential and the release of mitochondrial cell-death mediators by a Nur77-dependent pathway. J Cell Sci (2005) 118(Pt 19):4485–93. 10.1242/jcs.02580 16179607

[7] Chaabane W Cieslar-Pobuda A El-Gazzah M Jain MV Rzeszowska-Wolny J Rafat M. Human-gyrovirus-Apoptin triggers mitochondrial death pathway–Nur77 is required for apoptosis triggering. Neoplasia (2014) 16(9):679–93. 10.1016/j.neo.2014.08.001 PMC4234882 25246270

[8] Birame BM Jigui W Fuxian Y Jiazeng S Zhili L Weiquan L. Potentiation of Apoptin-induced apoptosis by Cecropin B-like antibacterial peptide ABPs1 in human HeLa cervical cancer cell lines is associated with membrane pore formation and caspase-3 activation. J Microbiol Biotechnol (2014) 24(6):756–64. 10.4014/jmb.1209.09076 24633228

[9] Rutkowski DT Arnold SM Miller CN Wu J Li J Gunnison KM. Adaptation to ER stress is mediated by differential stabilities of pro-survival and pro-apoptotic mRNAs and proteins. PloS Biol (2006) 4(11):e374. 10.1371/journal.pbio.0040374 17090218 PMC1634883

[10] Gong J Wang XZ Wang T Chen JJ Xie XY Hu H. Molecular signal networks and regulating mechanisms of the unfolded protein response. J Zhejiang Univ Sci B (2017) 18(1):1–14. 10.1631/jzus.B1600043 28070992 PMC5260473

[11] Luo X Cheng C Tan Z Li N Tang M Yang L. Emerging roles of lipid metabolism in cancer metastasis. Mol Cancer (2017) 16(1):76. 10.1186/s12943-017-0646-3 28399876 PMC5387196

[12] Harding HP Zhang Y Ron D. Protein translation and folding are coupled by an endoplasmic-reticulum-resident kinase. Nature (1999) 397(6716):271–4. 10.1038/16729 9930704

[13] Harding HP Zeng H Zhang Y Jungries R Chung P Plesken H. Diabetes mellitus and exocrine pancreatic dysfunction in perk-/- mice reveals a role for translational control in secretory cell survival. Mol Cell (2001) 7(6):1153–63. 10.1016/s1097-2765(01)00264-7 11430819

[14] Shi Y Vattem KM Sood R An J Liang J Stramm L. Identification and characterization of pancreatic eukaryotic initiation factor 2 alpha-subunit kinase, PEK, involved in translational control. Mol Cell Biol (1998) 18(12):7499–509. 10.1128/mcb.18.12.7499 PMC109330 9819435

[15] Rajagopalan S Xu Y Brenner MB. Retention of unassembled components of integral membrane proteins by calnexin. Science (1994) 263(5145):387–90. 10.1126/science.8278814 8278814

[16] Bergeron JJ Brenner MB Thomas DY Williams DB. Calnexin: a membrane-bound chaperone of the endoplasmic reticulum. Trends Biochem Sci (1994) 19(3):124–8. 10.1016/0968-0004(94)90205-4 8203019

[17] Williams DB. Beyond lectins: the calnexin/calreticulin chaperone system of the endoplasmic reticulum. J Cell Sci (2006) 119(Pt 4):615–23. 10.1242/jcs.02856 16467570

[18] Tu BP Ho-Schleyer SC Travers KJ Weissman JS. Biochemical basis of oxidative protein folding in the endoplasmic reticulum. Science (2000) 290(5496):1571–4. 10.1126/science.290.5496.1571 11090354

[19] Huppa JB Ploegh HL. The eS-Sence of -SH in the ER. Cell (1998) 92(2):145–8. 10.1016/s0092-8674(00)80907-1 9458037

[20] Frand AR Kaiser CA. The ERO1 gene of yeast is required for oxidation of protein dithiols in the endoplasmic reticulum. Mol Cell (1998) 1(2):161–70. 10.1016/s1097-2765(00)80017-9 9659913

[21] Tu BP Weissman JS. Oxidative protein folding in eukaryotes: mechanisms and consequences. J Cell Biol (2004) 164(3):341–6. 10.1083/jcb.200311055 PMC2172237 14757749

[22] Ellgaard L Ruddock LW. The human protein disulphide isomerase family: substrate interactions and functional properties. EMBO Rep (2005) 6(1):28–32. 10.1038/sj.embor.7400311 15643448 PMC1299221

[23] Cox JS Shamu CE Walter P. Transcriptional induction of genes encoding endoplasmic reticulum resident proteins requires a transmembrane protein kinase. Cell (1993) 73(6):1197–206. 10.1016/0092-8674(93)90648-a 8513503

[24] Hollien J Weissman JS. Decay of endoplasmic reticulum-localized mRNAs during the unfolded protein response. Science (2006) 313(5783):104–7. 10.1126/science.1129631 16825573

[25] Lee K Tirasophon W Shen X Michalak M Prywes R Okada T. IRE1-mediated unconventional mRNA splicing and S2P-mediated ATF6 cleavage merge to regulate XBP1 in signaling the unfolded protein response. Genes Dev (2002) 16(4):452–66. 10.1101/gad.964702 PMC155339 11850408

[26] Kohno K Normington K Sambrook J Gething MJ Mori K. The promoter region of the yeast KAR2 (BiP) gene contains a regulatory domain that responds to the presence of unfolded proteins in the endoplasmic reticulum. Mol Cell Biol (1993) 13(2):877–90. 10.1128/mcb.13.2.877 PMC358971 8423809

[27] Munro S Pelham HR. An Hsp70-like protein in the ER: identity with the 78 kd glucose-regulated protein and immunoglobulin heavy chain binding protein. Cell (1986) 46(2):291–300. 10.1016/0092-8674(86)90746-4 3087629

[28] Haas IG Wabl M. Immunoglobulin heavy chain binding protein. Nature (1983) 306(5941):387–9. 10.1038/306387a0 6417546

[29] Hetz C Chevet E Harding HP. Targeting the unfolded protein response in disease. Nat Rev Drug Discov (2013) 12(9):703–19. 10.1038/nrd3976 23989796

[30] Kawamura T Kanno R Fujii H Suzuki T. Expression of liver-type fatty-acid-binding protein, fatty acid synthase and vascular endothelial growth factor in human lung carcinoma. Pathobiology (2005) 72(5):233–40. 10.1159/000089417 16374067

[31] Shah US Dhir R Gollin SM Chandran UR Lewis D Acquafondata M. Fatty acid synthase gene overexpression and copy number gain in prostate adenocarcinoma. Hum Pathol (2006) 37(4):401–9. 10.1016/j.humpath.2005.11.022 16564913

[32] Wakil SJ Abu-Elheiga LA. Fatty acid metabolism: target for metabolic syndrome. J Lipid Res (2009) 50 Suppl:S138–43. 10.1194/jlr.R800079-JLR200 PMC2674721 19047759

[33] Fullerton MD Galic S Marcinko K Sikkema S Pulinilkunnil T Chen ZP. Single phosphorylation sites in Acc1 and Acc2 regulate lipid homeostasis and the insulin-sensitizing effects of metformin. Nat Med (2013) 19(12):1649–54. 10.1038/nm.3372 PMC4965268 24185692

[34] Migita T Narita T Nomura K Miyagi E Inazuka F Matsuura M. ATP citrate lyase: activation and therapeutic implications in non-small cell lung cancer. Cancer Res (2008) 68(20):8547–54. 10.1158/0008-5472.CAN-08-1235 18922930

[35] Towle HC Kaytor EN Shih HM. Regulation of the expression of lipogenic enzyme genes by carbohydrate. Annu Rev Nutr (1997) 17:405–33. 10.1146/annurev.nutr.17.1.405 9240934

[36] Kim Y Han JM Han BR Lee KA Kim JH Lee BD. Phospholipase D1 is phosphorylated and activated by protein kinase C in caveolin-enriched microdomains within the plasma membrane. J Biol Chem (2000) 275(18):13621–7. 10.1074/jbc.275.18.13621 10788479

[37] Gomez-Cambronero J. Phospholipase D in cell signaling: from a myriad of cell functions to cancer growth and metastasis. J Biol Chem (2014) 289(33):22557–66. 10.1074/jbc.R114.574152 PMC4132763 24990944

[38] Henkels KM Boivin GP Dudley ES Berberich SJ Gomez-Cambronero J Phospholipase D. (PLD) drives cell invasion, tumor growth and metastasis in a human breast cancer xenograph model. Oncogene (2013) 32(49):5551–62. 10.1038/onc.2013.207 PMC3966651 23752189

[39] Kato H Sakaki K Mihara K. Ubiquitin-proteasome-dependent degradation of mammalian ER stearoyl-CoA desaturase. J Cell Sci (2006) 119(Pt 11):2342–53. 10.1242/jcs.02951 16723740

[40] Ntambi JM Miyazaki M Stoehr JP Lan H Kendziorski CM Yandell BS. Loss of stearoyl-CoA desaturase-1 function protects mice against adiposity. Proc Natl Acad Sci U S A (2002) 99(17):11482–6. 10.1073/pnas.132384699 PMC123282 12177411

[41] Hotamisligil GS. Endoplasmic reticulum stress and the inflammatory basis of metabolic disease. Cell (2010) 140(6):900–17. 10.1016/j.cell.2010.02.034 PMC2887297 20303879

[42] Kumashiro N Erion DM Zhang D Kahn M Beddow SA Chu X. Cellular mechanism of insulin resistance in nonalcoholic fatty liver disease. Proc Natl Acad Sci U S A (2011) 108(39):16381–5. 10.1073/pnas.1113359108 PMC3182681 21930939

[43] Ozcan U Cao Q Yilmaz E Lee AH Iwakoshi NN Ozdelen E. Endoplasmic reticulum stress links obesity, insulin action, and type 2 diabetes. Science (2004) 306(5695):457–61. 10.1126/science.1103160 15486293

[44] Bobrovnikova-Marjon E Hatzivassiliou G Grigoriadou C Romero M Cavener DR Thompson CB. PERK-dependent regulation of lipogenesis during mouse mammary gland development and adipocyte differentiation. Proc Natl Acad Sci U S A (2008) 105(42):16314–9. 10.1073/pnas.0808517105 PMC2570995 18852460

[45] Jiang L Wang H Li J Fang X Pan H Yuan X. Up-regulated FASN expression promotes transcoelomic metastasis of ovarian cancer cell through epithelial-mesenchymal transition. Int J Mol Sci (2014) 15(7):11539–54. 10.3390/ijms150711539 PMC4139798 24979135

[46] Wang H Xi Q Wu G. Fatty acid synthase regulates invasion and metastasis of colorectal cancer via Wnt signaling pathway. Cancer Med (2016) 5(7):1599–606. 10.1002/cam4.711 PMC4864275 27139420

[47] Ahmad I Mui E Galbraith L Patel R Tan EH Salji M. Sleeping Beauty screen reveals Pparg activation in metastatic prostate cancer. Proc Natl Acad Sci U S A (2016) 113(29):8290–5. 10.1073/pnas.1601571113 PMC4961202 27357679

[48] Su YW Lin YH Pai MH Lo AC Lee YC Fang IC. Association between phosphorylated AMP-activated protein kinase and acetyl-CoA carboxylase expression and outcome in patients with squamous cell carcinoma of the head and neck. PloS One (2014) 9(4):e96183. 10.1371/journal.pone.0096183 24769813 PMC4000216

[49] Scaglia N Chisholm JW Igal RA. Inhibition of stearoylCoA desaturase-1 inactivates acetyl-CoA carboxylase and impairs proliferation in cancer cells: role of AMPK. PloS One (2009) 4(8):e6812. 10.1371/journal.pone.0006812 19710915 PMC2728543

[50] Wang H Zhang Y Lu Y Song J Huang M Zhang J. The role of stearoyl-coenzyme A desaturase 1 in clear cell renal cell carcinoma. Tumour Biol (2016) 37(1):479–89. 10.1007/s13277-015-3451-x 26224474

[51] Lee HS Park SY Lee HW Choi HS. Secretions of MMP-9 by soluble glucocorticoid-induced tumor necrosis factor receptor (sGITR) mediated by protein kinase C (PKC) delta and phospholipase D (PLD) in murine macrophage. J Cell Biochem (2004) 92(3):481–90. 10.1002/jcb.20099 15156560

